# Dorsal Medial Habenula Regulation of Mood-Related Behaviors and Primary Reinforcement by Tachykinin-Expressing Habenula Neurons

**DOI:** 10.1523/ENEURO.0109-16.2016

**Published:** 2016-07-18

**Authors:** Yun-Wei A. Hsu, Glenn Morton, Elizabeth G. Guy, Si D. Wang, Eric E. Turner

**Affiliations:** Center for Integrative Brain Research, Seattle Children’s Research Institute, Seattle, Washington 98101

**Keywords:** fear conditioning, habenula, interpeduncular nucleus, learned helplessness, substance P

## Abstract

Animal models have been developed to investigate aspects of stress, anxiety, and depression, but our understanding of the circuitry underlying these models remains incomplete. Prior studies of the habenula, a poorly understood nucleus in the dorsal diencephalon, suggest that projections to the medial habenula (MHb) regulate fear and anxiety responses, whereas the lateral habenula (LHb) is involved in the expression of learned helplessness, a model of depression. Tissue-specific deletion of the transcription factor Pou4f1 in the dorsal MHb (dMHb) results in a developmental lesion of this subnucleus. These dMHb-ablated mice show deficits in voluntary exercise, a possible correlate of depression. Here we explore the role of the dMHb in mood-related behaviors and intrinsic reinforcement. Lesions of the dMHb do not elicit changes in contextual conditioned fear. However, dMHb-lesioned mice exhibit shorter immobility time in the tail suspension test, another model of depression. dMHb-lesioned mice also display increased vulnerability to the induction of learned helplessness. However, this effect is not due specifically to the dMHb lesion, but appears to result from *Pou4f1* haploinsufficiency elsewhere in the nervous system. *Pou4f1* haploinsufficiency does not produce the other phenotypes associated with dMHb lesions. Using optogenetic intracranial self-stimulation, intrinsic reinforcement by the dMHb can be mapped to a specific population of neurokinin-expressing habenula neurons. Together, our data show that the dMHb is involved in the regulation of multiple mood-related behaviors, but also support the idea that these behaviors do not reflect a single functional pathway.

## Significance Statement

Our current understanding of neural circuits regulating mood states, such as fear and depression, is fragmentary. Recently, interest has grown in how the habenula, a poorly understood nucleus providing descending inputs to the tegmentum and raphe, may affect these behavioral states. We have used mouse genetic models to study part of this system, the dorsal medial habenula (dMHb). Here we report that the dMHb is not, as previously proposed, required for normal acquisition of a conditioned fear response. Mice with genetic lesions of the dMHb show some profound effects in mood-related tests, but cannot be described strictly as “depressed” or “resilient”, suggesting that at the circuit level, models of affective states are complex and do not report identical phenomena.

## Introduction

The habenula is a poorly understood brain nucleus lying dorsal to the thalamus. Recently, a series of studies has begun to explore the role of the habenula in behaviors related to mood regulation and stress. The habenula is divided into lateral habenula (LHb) and medial habenula (MHb) subnuclei, and the MHb can be further subdivided into a dorsal part (dMHb), containing excitatory neurons characterized by the expression of the tachykinin gene *Tac1*, encoding the neuropeptide substance P (SP), and a ventral part (vMHb), which contains glutamatergic neurons that also produce acetylcholine (Quina et al., 2009; Hsu et al., 2013). Anatomical studies have demonstrated that these subnuclei have distinct neural inputs and downstream targets in the brainstem. Past experiments have generally performed lesions of the entire habenula or its major output tract, the fasciculus retroflexus, and have assigned a wide variety of behavioral functions to the habenula (Lecourtier and Kelly, 2007), but this approach cannot resolve the specific functions of the habenula subnuclei.

Recent experiments targeting the specific input pathways from the septal nuclei to the vMHb and dMHb have implicated these circuits in the regulation of anxiety and fear responses, respectively (Yamaguchi et al., 2013). Nonetheless, it remains to be determined whether the MHb subnuclei are directly involved in the expression of anxiety and fear responses. A pathway through the LHb has recently been shown to be involved in the expression of learned helplessness (B. Li et al., 2011; K. Li et al., 2013), a model of depression in which animals previously exposed to an inescapable aversive stimulus show diminished escape behavior when the stimulus is avoidable (Maier, 1984; Maier and Watkins, 2005; Duman, 2010). The LHb has well established roles in reward and aversive functioning (Hikosaka, 2010; Proulx et al., 2014). The role of the LHb in mediating depression-like behaviors may thus be related to its roles in punishment (Matsumoto and Hikosaka, 2009), and aversion (Lammel et al., 2012; Stamatakis and Stuber, 2012). Although the MHb has distinct input and output circuitry from the LHb, recent evidence has shown that the MHb may also play a role in regulation of mood-related behaviors. Recently we have shown that mice with a developmental ablation of the dMHb, generated by targeted deletion of the transcription factor *Pou4f1* (dMHb^CKO^ mice), exhibit reduced voluntary wheel-running activity (WRA), a possible correlate of depression (Hsu et al., 2014). In addition, in contrast to the aversive effect of LHb stimulation, the dMHb is intrinsically reinforcing in a self-stimulation paradigm (Hsu et al., 2014), lending additional support for a role for the dMHb in the maintenance of hedonic states.

In the present study, we investigated whether neurons in the dMHb regulate mood-related behaviors by testing dMHb^CKO^ mice in models of fear (conditioned fear) and depression (tail suspension test, learned helplessness). We found that mice with dMHb lesions do not exhibit differences in contextual conditioned fear. The effects of dMHb ablation in models of depression is significant, but cannot be interpreted simply as “depressed” or “resilient”. Using a Cre-driver line that is specific for a tachykinin-expressing subpopulation of dMHb neurons, we show that activation of these neurons is sufficient to support self-stimulation reinforcement. Collectively, our data show that the dMHb is involved in the regulation of multiple mood-related behaviors, but also suggest that these behaviors are not mediated by a single neural pathway.

## Materials and Methods

### Transgenic mice used in the experiments

**Figure 1.**
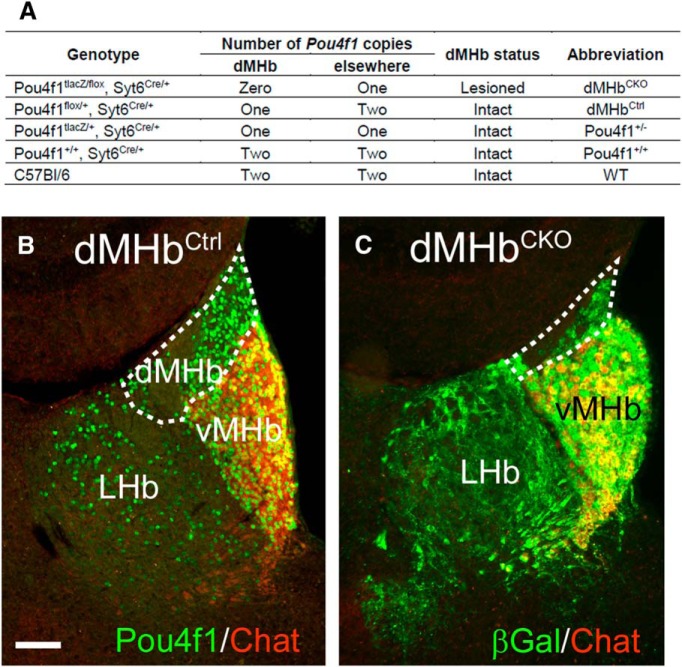
*Pou4f1* knock-out models used for analysis of dMHb function. A, Summary of genetic models used to generate dMHb lesions. ***B***, ***C***, Coronal sections through the habenula at bregma −1.58mm were stained with antibodies for choline acetyltransferase (Chat) and either Pou4f1 (Brn3a protein) to reveal habenula neurons expressing this factor, or for the *lacZ* gene product βGal, expressed by the *Pou4f1^tlacZ^* allele, which allows the identification of neurons that would normally express Pou4f1 in cells in which the gene has been deleted. ***B***, dMHb^Ctrl^ mouse with the genotype *Pou4f1^flox/+^/Syt6^Cre^*. Nuclear staining for Pou4f1 shows expression in both the vMHb and dMHb, and scattered expression in the LHb. The vMHb is distinguished by the expression of Chat, and the Pou4f1-positive, Chat-negative dMHb is circled. Scale bar, 100 μm. ***C***, dMHb^CKO^ mouse with the genotype *Pou4f1^flox/tlacZ^/Syt6^Cre^*. Staining for *lacZ* is used to show the extent of the dMHb lesion in the absence of Pou4f1 protein. The extent of the dMHb is greatly reduced (circle) and only a few βGal-positive, Chat-negative neurons remain in the medial habenula. Neurons of the vMHb, distinguished by Chat expression in ***A*** and ***B***, are intact in both the dMHb^Ctrl^ and dMHb^CKO^ mice. The area within the dMHb that is not stained by any of the antibodies used consists of axon tracts of the striae medularis and/or habenula commissure.

Mice with a tissue-specific null mutation of *Pou4f1* in the dMHb and control littermates used in this study were generated and genotyped as previously described (Hsu et al., 2014). The *Pou4f1* mutant mice used and their patterns of Pou4f1 transcription factor expression are listed in Figure 1*A*. Two strains of *Pou4f1* mutant mice were used: *Pou4f1^tlacZ^*, a functionally null allele replacing the *Pou4f1* coding sequence with a β-galactosidase expression cassette (Quina et al., 2005; MGI:3512089), *Pou4f1^flox^*, in which the principal coding exon of *Pou4f1* is flanked by loxP sequences (Hsu et al., 2014, MGI:5662420). The *Pou4f1^flox^* allele was excised using a Cre-recombinase expressing line, Syt6^Cre^, (STOCK Tg(Syt6-Cre)KI148Gsat/Mmcd), a BAC transgenic generated by the GENSAT project (Gerfen et al., 2013; RRID:MMRRC_032012-UCD), and obtained as cryopreserved sperm from the Mutant Mouse Regional Resource Center of the University of California, Davis. Experimental mice with dMHb lesions and littermate controls were generated by crossing mice with the genotype *Pou4f1^tlacZ/+^, Syt6^Cre/Cre^* with *Pou4f1^flox/flox^* mice to yield the genotypes *Pou4f1^tlacZ/flox^, Syt6^Cre/+^* (dMHb^CKO^) and *Pou4f1^flox/+^, Syt6^Cre/+^* (dMHb^Ctrl^) mice in equal ratios. The constitutive null *Pou4f1^tlacZ^* allele was used to generate dMHb^CKO^ mice because the generation of animals with complete loss of Pou4f1 expression in the dMHb is more efficient if one allele is a constitutive null, and thus only one copy of the gene requires Cre-excision The presence of the *lacZ* gene product βGal also allows dMHb neurons to be identified by enzymatic staining or immunofluorescence in cells which no longer express Pou4f1 protein. The dMHb^CKO^ mice show a profound loss of neurons in the dMHb due to postnatal cell death, whereas dMHb^Ctrl^ mice do not show detectable loss of dMHb neurons (Fig. 1*B*,*C*).

In order to isolate an effect of *Pou4f1* haploinsufficiency in the presence of an intact dMHb, mice that were homozygous or hemizygous for *Pou4f1* were generated by crossing male mice with the genotype *Pou4f1^tlacZ/+^, Syt6^Cre/Cre^* with C57Bl/6 female mice to yield the genotypes *Pou4f1^tlacZ/+^, Syt6^Cre/+^* (Pou4f1^+/-^) and *Pou4f1^+/+^, Syt6^Cre/+^* (Pou4f1^+/+^) mice in equal ratios. In these genotypes, the *Syt6^Cre^* allele has no effect because no floxed allele is present, but *Syt6^Cre^* was incorporated to maintain a consistent genetic background across all lines.

Mice for optogenetic studies of habenula function were generated by using the mouse line *Ai32*, which conditionally expresses the Channelrhodopsin-2 variant ChR2(H134R)-YFP from a modified floxed-stop Gt(Rosa)26Sor locus (Madisen et al., 2012; RRID:IMSR_JAX:024109). To generate mice expressing ChR2 in tachykinin-expressing neurons in the dMHb (dMHb^ChR2^ mice), *Ai32* mice were interbred with mice bearing a *Tac2^IRESCre^* allele, generated as a part of the Allen Institute Transgenic Characterization Project (Harris et al., 2014; RRID:IMSR_JAX:021878). Experimental mice from these crosses had the genotype *Tac2^IRESCre^*/*Ai32*, and control mice bore the *Ai32* allele, without *Tac2^IRESCre^*. Mice expressing ChR2 in the vMHb (vMHb^ChR2^ mice) were generated by interbreeding *Ai32* mice with mice bearing a *Chat^IRESCre^* allele (Rossi et al., 2011; RRID:IMSR_JAX:006410) and have been previously described (Hsu et al., 2013). *Tac2^IRESCre^* and *Chat^IRESCre^* mice were obtained from Jackson Laboratories. Only male mice were used for the optogenetic experiments.

### Analysis of gene expression

Immunofluorescence methods, including antibodies used to detect the *Pou4f1* protein Brn3a (RRID: AB_2314040), choline acetyltransferase (RRID: AB_2079751), substance P (RRID:AB_94639), and β-galactosidase (not in RRID), have been previously described (Hsu et al., 2014). Rabbit anti-mouse proNeurokininB was obtained from Novus Biologicals (RRID:AB_350516).

### Contextual conditioned fear

Fear conditioning was performed in a 13 × 17 cm compartment (1 compartment of ENV-3013, Med Associates, equipped with grid floor ENV-3013BR). The grid floor was connected to a shock scrambler (ENV-414S, Med Associates) set to 0.40 mA. The Activity Tracking function of Noldus EthoVision XT 10 was used to analyze freezing behavior, which is defined by the absence of observable movement, except for respiration. On day 1, mice were allowed to acclimate to the chamber for 3 min, followed by the delivery of a 1 s shock every minute for 6 min. To the extent possible, this training procedure was designed to reproduce the methods used in a prior study (Masugi et al., 1999; Yamaguchi et al., 2013). On day 2 of the procedure, animals were returned to the same compartment for 6 min in the absence of shocks in order to assess the contextual conditioned fear response.

We implemented automated scoring of freezing behavior based on a published method using an earlier version of Noldus EthoVision (Pham et al., 2009). Automated analysis of the video derived the image of the subject in each video frame (frame rate 30 s^−1^, duration 33.33 ms). A moving average of the subject image in the previous three video frames was then compared to the current frame, and the subject was determined to be immobile if the image area occupied by the subject in the current frame was changed (displaced, or altered in size or shape) from the moving average by <0.02%. The activity was scored as freezing if immobility lasted longer than 0.5 s, corresponding to 15 consecutive samples of immobility at the video frame rate of 30 s^−1^. Reliability of the automated video analysis of freezing behavior was confirmed by manual scoring of test videos of control mice by two human raters blind to the automated scoring results.

### Tail suspension test

The test was performed using inverted U-shaped acrylic stand (18 × 18 inches, with a 4 inch wide horizontal arm), placed in an isolation chamber with a video camera positioned to include 1.5 inches of the horizontal arm and the entire length of the subject within the field-of-view. At the start of the trial, subjects were suspended from the crossbar using tape placed one inch from the tip of the tail. Although C57BL/6 mice have been observed to climb their tails when performing this test, this behavior was not observed using this apparatus. A 6 min video was recorded and analyzed in Noldus EthoVision XT 10.0 using Activity Tracking. Each recorded video frame was compared to the previous frame (frame rate 30 s^−1^, duration 33.33 ms) and the mouse was determined to be immobile for that period if <5% change was observed from one frame to the next.

### Learned helplessness and active avoidance

The learned helplessness protocol consisted of a training session or sessions in which mice were exposed to inescapable shock stimuli, followed by analysis of the learned helplessness response using active avoidance in a two-way shuttle box. Training consisted of 360 shocks of random duration between 1 and 3 s and random interstimulus interval of 1–15 s, delivered over ∼1 h, using Noldus EthoVision XT 10.0. Active avoidance was assessed using 60 escape trials on the following day, or after 3 weeks to assess the persistence of the learned helplessness response.

Learned helplessness training was performed in a 13 × 17 cm chamber (either of the main compartments of ENV-3013, Med Associates). Chambers were equipped with conductive grid and rod floors connected to a shock scrambler (ENV-414S, Med Associates) set to 0.40 mA. The active avoidance test was performed in a 42 × 16.5 cm center channel modular shuttle box chamber (ENV-010MC, Med Associates) equipped with a video monitoring and analysis system (Noldus EthoVision XT 10.0). The grid floor was connected to the same scrambler used for training and set to 0.40 mA. Sixty escape trials were performed, each consisting of a 4.5 kHz tone followed by a shock. The tone preceded the onset of the shock by 2 s and continued until the shock event stopped. The maximum duration of each shock was 4 s, and the shock could be avoided by crossing to the opposite side of the compartment during the tone, or stopped by crossing during the shock. A random intertrial interval of 19–29 s was used between trials. Latency to escape was scored as 0 if the animal successfully escaped during the tone before onset of the shock. Otherwise, latency time was calculated from shock onset to the time of escape. A maximum score of 4 was given if the animal failed to escape the shock. The mean latency to escape was calculated over the 60 trials for each mouse.

Over the course of the experiments, we observed that a small number of mice adopted an unintended strategy for shock avoidance in which they straddled the midline of the shuttle box. This behavior caused the video analysis to dither and falsely report a very high frequency of midline crossings, which was easily identified on analysis of the data. The shuttle box arenas defined in the video analysis software were modified in later experiments such that the mouse was required to travel at least 4 cm beyond the midline in order to avoid or terminate the shock. This modification somewhat increased escape latency because of the increased distance traveled. Cohorts of 10 C56Bl/6 male mice were used to assess the effect of this change in the arena boundaries on the measurement of the learned helplessness response. Significant differences were observed between trained and naïve animals using both arena structures.

### Accelerating rotarod

A specialized apparatus (Rotamex-5, Columbus Instruments) was used to test rotarod performance as previously described (Hsu et al., 2014). Briefly, before testing all mice were trained on a fixed-speed protocol at 4 rpm until they could stay on the rod for 30 s. On the same day as the training sessions, mice underwent four 5 min accelerating rotarod trials. In each trial, the rotarod accelerated from 4 to 40 rpm at the rate of 1 rpm every 8 s, then remained at 40 rpm until the end of the trial. The principal outcome was the time (latency) until the mouse fell from the rod. Mice were given at least 15 min of rest in between each trial. To calculate the average latency to fall for each mouse, the lowest of four values was discarded.

### Optogenetic intracranial self-stimulation

Intracranial self-stimulation (ICSS) reinforcement by the dMHb was tested in an operant chamber (ENV-307W, Med Associates) equipped with two nosepoke receptacles (ENV-313W, Med Associates). Responses were recorded through four training and four reversal sessions.

#### Training sessions

Mice underwent four 45 min ICSS sessions in which a randomly assigned active nose-poke receptacle was associated with the delivery of laser stimulation to the dMHb via an implanted fiber optic cannula (above), and the inactive receptacle did not deliver a stimulus. A 1:1 fixed response–reward ratio was used. The reward consisted of a 2 s train of 25 ms light pulses, delivered at 20 Hz and 8 mW with a 473 nm laser, followed by a 2 s time-out period. Nose pokes during the period of laser stimulation and time-out were recorded but did not trigger a reward. Nose pokes on the inactive receptacle were also recorded.

#### Reversal sessions

The reinforcing effect of ICSS was confirmed in four reversal sessions during which the active receptacle was switched to the opposite side, using the same session structure.

### Real-time place preference

Real-time place preference (RTPP) studies were conducted in a two-chamber place-preference box (ENV-010MC, Med Associates) in which mice received light stimulation in one side, and could move freely between compartments. Recording and laser stimulation were controlled with EthoVision XT using center point tracking. Sessions were initiated by placing the mouse in the center of the apparatus. The mice were then given free access to both chambers for 15 min. When the mouse entered the active chamber, the 473 nm laser was activated, delivering 25 ms light pulses at a 20 Hz and 8 mW of total power continuously until the mouse crossed over to the inactive chamber. Conversely, upon entering the inactive chamber, the laser remained off until the mouse entered the active chamber. Active and inactive chamber occupancy and total distance traveled were then calculated for each 5 min interval of the 15 min session.

### General statistical methods

Statistical analyses were conducted using unpaired two-tailed *t* tests, two-way ANOVA or two-way repeated-measures ANOVA, with Tukey’s or Bonferroni’s *post hoc* analyses in GraphPad Prism 6 (GraphPad Software). Results are presented as mean ± SEM. We also considered that in this paper and in a prior paper (Hsu et al., 2014), we compared dMHb^CKO^ mice and controls in multiple behavioral models that use a motor output measure as a model of depression/behavioral despair. These tests include learned helplessness, the persistence of learned helplessness, and the tail suspension test in the present study, and the forced swim test in the prior study. In the prior study, we also interpreted a deficit in rotarod function as possibly related to behavioral despair. Given that five independent tests were applied, Bonferroni correction for the number of tests suggests that a threshold significance value of *p*< 0.01 rather than *p*<0.05 should be used. Using this more stringent standard, the effect of genotype is still significant for learned helplessness, persistence of learned helplessness, tail suspension, and rotarod tests. The effect in the forced swim test was not significant at *p*<0.05.

## Results

### The conditioned fear response is not affected by developmental loss of dMHb neurons

**Figure 2.**
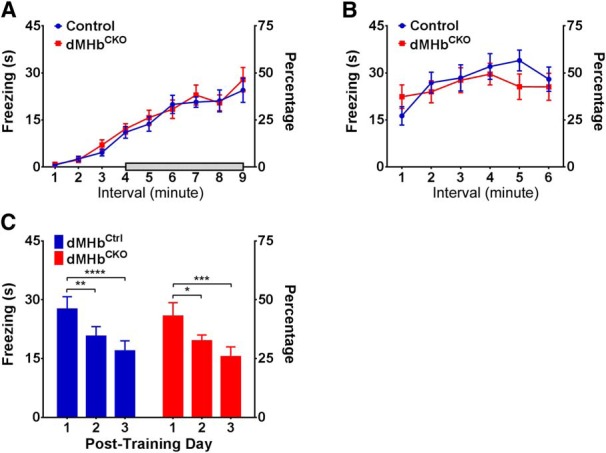
Conditioned fear response in dMHb^CKO^ mice. ***A***, Training session: time spent in freezing behavior in 1 min intervals during 3 min of acclimation, followed by six intervals preceded by the delivery of a 1 s shock, is shown. Shaded bar shows the period of shock administration. ***B***, Contextual conditioned fear response: on the testing day, the time spent in freezing behavior was assessed in the same environment as the training session, but without shock delivery, to evaluate the conditioned fear response. dMHb^CKO^ and dMHb^Ctrl^ mice exhibited the same amount of freezing during both the training and testing day. ***C***, Extinction of the conditioned fear response: time spent in freezing behavior during 3 subsequent days of testing is shown. The conditioned fear response showed gradual extinction in the absence of further shock stimuli. *p = 0.033, **p = 0.0082, ***p = 0.0003, and ****p < 0.0001, significant difference between days for these genotypes. *N* = 12 dMHbCtrl and 10 dMHb^CKO^ mice.

Recently, lesion studies of the specific septal inputs to the dorsal and ventral MHb have shown that the septohabenular pathway is involved in the regulation of anxiety and fear (Yamaguchi et al., 2013). Specifically, lesions of septal inputs to the dMHb increased freezing behavior in the training phase of a conditioned fear paradigm. (Contextual conditioned fear per se was not tested). To test for direct involvement of the dMHb in fear conditioning we assessed fear acquisition and the contextual fear response using dMHb^CKO^ mice and controls. The dMHb^CKO^ mouse bears a dMHb-specific deletion of the *Pou4f1* coding sequence, and shows a nearly complete developmental loss of dMHb neurons (Fig. 1). Both the dMHb^Ctrl^ and dMHb^CKO^ mice exhibited the same amount of freezing on the training day (day1) during shock administration (two-way ANOVA; genotype × trial, effect of genotype: *F*_(1,20)_ = 0.11, *p* = 0.74; Fig. 2*A*, time 4–9 min), and time spent in freezing increased with each subsequent shock administration (effect of trial: *F*_(5,100)_ = 18.48, *p* < 0.0001). Contextual fear response was tested on day 2 by placing the mice in the same environment without shock administration (Fig. 2*B*). Both genotypes exhibited the same amount of freezing (two-way ANOVA; effect of genotype: *F*_(1,20)_ = 0.15, *p* = 0.70), with increasing freezing behavior as the test progressed (effect of trial: *F*_(5,100)_ = 8.45, *p* < 0.0001). Finally, we examined the time course for the extinction of conditioned fear response. Fear extinction was tested over 3 subsequent days of exposure to the environment without shock stimuli (Fig. 2*C*). The freezing response gradually diminished in a similar manner for both genotypes (two-way ANOVA; effect of genotype: *F*_(1,20)_ = 0.20, *p* = 0.66; effect of day: *F*_(2,40)_ = 21.91, *p* < 0.0001). *Post hoc* analyses revealed that both the dMHb^Ctrl^ and dMHb^CKO^ mice spent significantly less time freezing starting at 2 d post-training. Thus, we conclude that dMHb ablation has no significant effect on acquisition of the fear response, and no effect on contextual conditioned fear or its extinction.

### *Pou4f1* gene dosage affects learned helplessness in an active avoidance model

Another model for assessing depression-like behavioral changes in rodents is learned helplessness. In this paradigm, animals are exposed to stress in the form of inescapable shocks, and the uncontrollability of the stressful stimulus is thought to generate a learned helplessness behavior in which animals subsequently fail to escape an aversive stimulus in a different environment (Maier, 1984). Like the forced swim test (FST) and tail suspension test (TST), learned helplessness has been used to predict antidepressant response (Duman, 2010). In the implementation of learned helplessness used here, mice were administered inescapable shocks during one training session in an apparatus used exclusively for the training. Mice were then tested 24 h later for active avoidance in a separate two-way shuttle box to assess the learned helplessness response (Chourbaji et al., 2005). The mice were also tested 3 weeks later to determine the persistence of learned helplessness.

**Figure 3.**
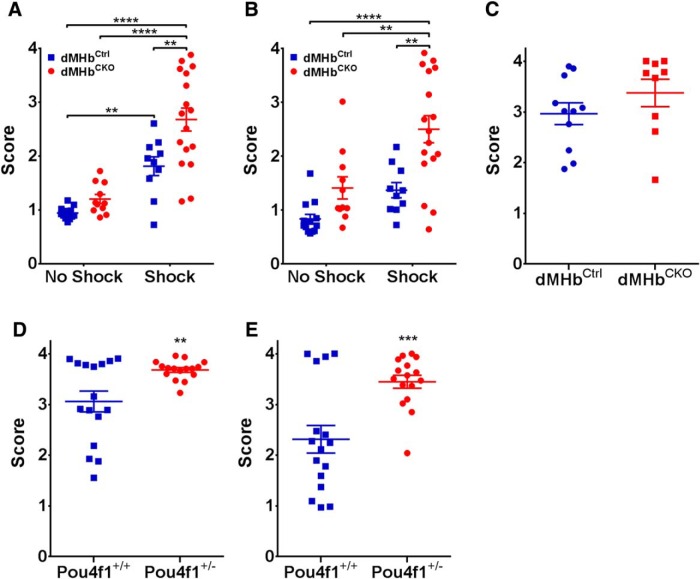
Learned helplessness assessed by active avoidance in dMHb^CKO^ mice. ***A***, Learned helplessness response: the mean latency to escape in the shuttle box following 1 d of inescapable shock training, or exposure to the training chamber without a shock, is shown. Both the dMHb^CKO^ and dMHb^Ctrl^ mice that received shocks during training showed increased latency to escape, but dMHb^CKO^ mice exhibited a stronger effect. **p < 0.01, ****p < 0.0001 for difference between groups indicated. ***B***, Persistence of the learned helplessness response: the mean latency to escape was reassessed for the cohort shown in ***A*** 3 weeks after inescapable shock training. dMHb^Ctrl^ mice that received inescapable shocks returned to near-baseline escape times. dMHb^CKO^ mice that received inescapable shocks retained the learned helplessness behavior. **p < 0.01 and ****p < 0.0001, significant difference between groups. *N* = 13 no-shock dMHbCtrl, 10 shocked dMHb^Ctrl^, 11 no-shock dMHb^CKO^, and 17 shocked dMHb^CKO^ mice. ***C***, Convergence of learned helplessness response with extended training: the mean latency to escape was assessed after 3 d of inescapable shock training in a different cohort of mice. Both the dMHb^CKO^ and dMHb^Ctrl^ mice received shocks during the training. The prolonged training increased escape latency for both genotypes when assessed by active avoidance 1 d after the final training session. *N* = 11 dMHb^Ctrl^ and 9 dMHb^CKO^ mice. ***D***, ***E***, Assessment of learned helplessness in a separate cohort of *Pou4f1* hemizygous mice. ***D***, Learned helplessness response: the mean latency to escape in the shuttle box following 1 d of inescapable shock training is shown. Both the Pou4f1^+/-^ and Pou4f1^+/+^ (wild-type) mice received shocks during the training. Pou4f1^+/-^ mice exhibited increased latency to escape relative to Pou4f1^+/+^ mice, i.e., were more susceptible to the induction of learned helplessness. **p = 0.0059, significant difference between the genotypes. ***E***, Persistence of learned helplessness response: the mean latency to escape was reassessed for the cohort shown in ***D*** 3 weeks after inescapable shock training. The Pou4f1^+/-^ mice showed persistently elevated escape latency relative to Pou4f1^+/+^ mice. ***p = 0.0007, significant difference between the genotypes. Minor modifications to the active avoidance protocol used in ***D*** and ***E***, resulting in somewhat longer escape times, are described in Materials and Methods.

Both dMHb^CKO^ and dMHb^Ctrl^ mice that received inescapable shocks during training took longer to escape the shocked compartment on the testing day compared to unshocked mice (two-way ANOVA; genotype × treatment, effect of treatment: *F*_(1,47)_ = 49.50, *p* < 0.0001; Fig. 3*A*), and mice also showed a significant difference in escape latency between genotypes (effect of genotype: *F*_(1,47)_ = 11.39, *p* = 0.0015). *Post hoc* analyses indicate that although no difference was observed between the dMHb^CKO^ and dMHb^Ctrl^ mice that did not receive inescapable shock training (*p* = 0.70), dMHb^CKO^ mice that received inescapable shocks had longer escape latency compared to shocked dMHb^Ctrl^ mice (*p* = 0.0029). Thus, the genetic lesion in dMHb^CKO^ mice rendered them more vulnerable to the development of learned helplessness behavior. The learned helplessness phenotype persisted 3 weeks after shock training (Fig. 3*B*), and there was a significant difference attributable to shock administration (*F*_(1,47)_ = 15.38, *p* = 0.0003) and genotype (*F*_(1,47)_ = 16.99, *p* = 0.0002). *Post hoc* analyses suggest that this is due to the persistence of learned helplessness response retained by the learned helplessness dMHb^CKO^ mice. We also examined the effect of extended training and determined that learned helplessness response of the dMHb^CKO^ and dMHb^Ctrl^ mice converged after 3 d of inescapable shock administration (*t*_(18)_ = 1.20, *p* = 0.24; Fig. 3*C*).

Although the most obvious explanation for the sensitization to learned helplessness in dMHb^CKO^ mice is the nearly complete loss of dMHb neurons in these animals, these mice possess one conditional *Pou4f1* allele (*Pou4f1^flox^*) and one *Pou4f1* null allele (*Pou4f1^tlacZ^*; see Materials and Methods), and are thus globally hemizygous for *Pou4f1*. To determine whether the observed learned helplessness phenotype resulted from the developmental loss of dMHb neurons, or could be a general effect of *Pou4f1* haploinsufficiency, we also examined learned helplessness in Pou4f1^+/-^ mice and Pou4f1^+/+^ controls. After 1 d of inescapable shock administration, Pou4f1^+/-^ mice took longer to escape the shocked compartment during the active avoidance test compared to Pou4f1^+/+^ mice (*t*_(30)_ = 2.97, *p* = 0.0059; Fig. 3*D*). As observed in the dMHb^CKO^ mice, the learned helplessness response persisted in the Pou4f1^+/-^ mice 3 weeks after the initial shock administration (*t*_(30)_ = 3.78, *p* = 0.0007; Fig. 3*E*). We conclude that the increased susceptibility to induction of learned helplessness behavior in Pou4f1-deficient mice is not exclusively dependent on the ablation of dMHb neurons.

### dMHb-lesioned mice exhibit prolonged escape behavior in the tail suspension test

**Figure 4.**
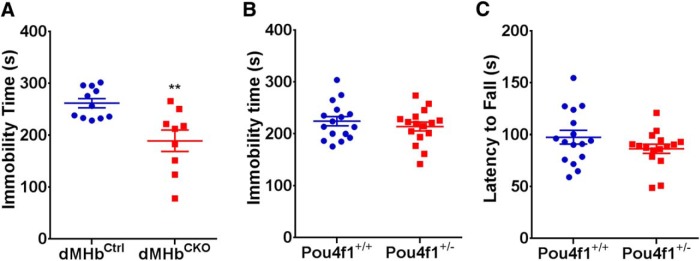
TST immobility time in dMHb^CKO^ and *Pou4f1* hemizygous mice, and affect of hemizygosity on rotarod performance. ***A***, Time spent immobile in the TST is shown for dMHb^CKO^ and dMHb^Ctrl^ mice. **p < 0.01, significance difference between the genotypes. *N* = 11 dMHbCtrl and 9 dMHb^CKO^ mice. ***B***, Time spent immobile in the TST is shown for Pou4f1^+/+^ and Pou4f1^+/-^ mice. *Pou4f1* gene dosage did not affect immobility time in the absence of a developmental dMHb lesion. *N* = 16 Pou4f1^+/+^ and 16 Pou4f1^+/-^ mice. Pou4f1^+/-^ mice have the genotype *Pou4f1^+/tlacZ^*. ***C***, Rotarod performance is not affected in Pou4f1^+/-^ mice. Both the Pou4f1^+/-^ and Pou4f1^+/+^ mice had similar latency to fall times in this test. *N* = 16 of each genotype.

Previously reported experiments have shown that developmental loss of the dMHb does not increase immobility time during the forced swim test, a model of stress response and depression (Duman, 2010; Hsu et al., 2014). Here, we assessed the behavior of dMHb^CKO^ mice using another model of depression, the TST (Steru et al., 1985; Cryan et al., 2005). Rodents suspended by their tails will initially struggle to escape, but eventually stop and become immobile. The time that the mice spend immobile in a trial of fixed duration is recorded as the principal outcome of the test. dMHb^CKO^ mice showed lower immobility time compared to dMHb^Ctrl^ mice during the 6 min test (*t*_(18)_ = 3.47, *p* = 0.0027; Fig. 4*A*).

To test whether the TST phenotype could be due to haploinsufficiency of *Pou4f1*, rather than the dMHb lesion observed in dMHb^CKO^ mice, we also performed the TST on cohorts of matched Pou4f1^+/-^ and Pou4f1^+/+^ mice (Fig. 4*B*). No difference in TST immobility time was observed between Pou4f1^+/-^ and Pou4f1^+/+^ mice (*t*_(30)_ = 0.84, *p* = 0.41). Thus, we conclude that the developmental loss of the dMHb mediates the reduced TST immobility observed in dMHb^CKO^ mice.

### Pou4f1 haploinsufficiency does not affect rotarod and voluntary wheel running performance

A prior study has shown that dMHb^CKO^ mice have a deficit in the accelerating rotarod test, with a markedly shortened latency to fall from the device, and a reduction in voluntary WRA (Hsu et al., 2014). Although the rotarod is usually used to assess motor deficits, other tests of motor function in these mice, including open-field locomotion, gait, and balance beam performance, were largely normal (Hsu et al., 2014). For this reason, it was concluded that the deficits in rotarod and WRA in dMHb^CKO^ mice likely resulted from a loss of motivation or reinforcement, rather than a deficit in motor function per se. Here, however, we have shown that *Pou4f1* haploinsufficiency can contribute to a mood-related phenotype, learned helplessness. Thus, to determine whether *Pou4f1* haploinsufficiency affects rotarod performance, we repeated this test in Pou4f1^+/-^ and Pou4f1^+/+^ mice. Both genotypes have similar fall latency in the rotarod test (*t* test, *t*_(30)_ = 1.42, *p* = 0.17; Fig. 4*C*), confirming that the reported deficit in rotarod performance in dMHb^CKO^ mice results from the developmental loss of dMHb neurons, not from *Pou4f1* haploinsufficiency.

Experiments to be published elsewhere demonstrate that WRA does not differ significantly between Pou4f1^+/-^ and Pou4f1^+/+^ mice (Y.-W. Hsu, H. de la Iglesia and E. Turner unpublished observations), and thus that the WRA deficit in dMHb^CKO^ mice is also specifically related to dMHb ablation.

### Tachykinin-expressing dMHb neurons mediate ICSS reinforcement

**Figure 5.**
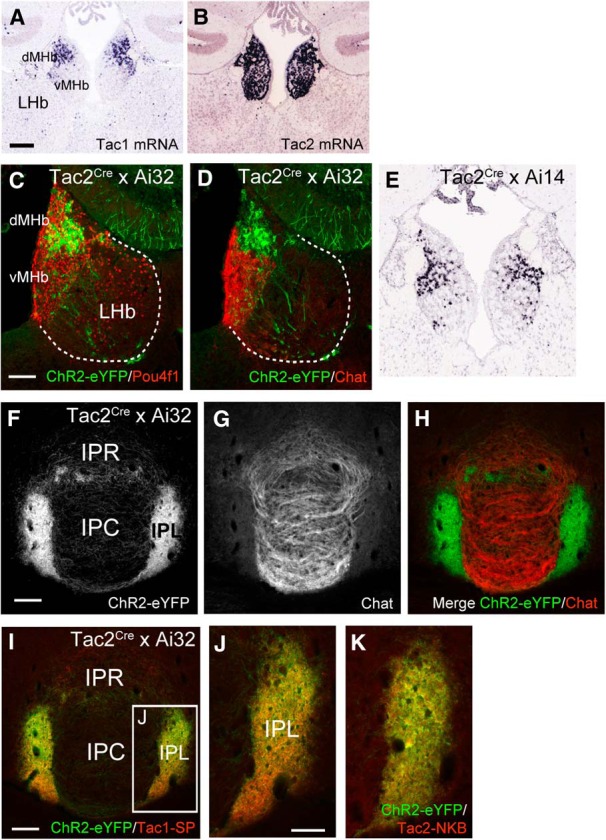
Specific expression of channelrhodopsin in tachykinin-expressing neurons in dMHb-Tac^ChR2^ mice. ***A***, ***B***, Comparison of Tac1 and Tac2 mRNA expression in the habenula; the axial level is approximately bregma −1.6mm. Data are derived from the Allen Mouse Brain Atlas. ***C***, ***D***, Conditional expression of ChR2-eYFP in *Ai32* mice driven by *Tac2^IRESCre^* in the habenula. Dashed lines demarcate the extent of the habenula based on the expression of the nuclear factor Pou4f1 (***C***). Expression is infrequently seen in the vMHb, as defined by the expression of Chat (***D***). ***E***, Conditional expression of tdTomato mRNA in Ai14 mice driven by *Tac2^IRESCre^* in the habenula (Allen Transgenic Mouse Characterization Project). ***F*–*H***, ChR2-eYFP labeled fibers terminate predominantly in the lateral part of the interpeduncular nucleus, which receives afferents from the dMHb (***F***, ***H***); these fibers are largely excluded from the IPR/IPC, which receive fibers mainly from the vMHb (***G***). ***I***, ***J***, Colocalization of SP, product of the *Tac1* gene, with ChR2-eYFP in the interpeduncular nucleus of dMHb-Tac^ChR2^ mice. ***K***, Colocalization of neurokinin B, product of the *Tac2* gene, with ChR2-eYFP. Scale bars: ***A***, 200 μm; ***C***, ***F***, ***I***, 100 μm; ***J***, 50 μm.

The dMHb is characterized by the expression of the tachykinin genes *Tac1* (encoding SP), expressed exclusively in this subnucleus (Fig. 5*A*; Quina et al., 2009), and *Tac2* (encoding NKB), which is expressed in the dMHb and vMHb (Fig. *5B*). Here we wished to test whether these tachykinin-expressing dMHb neurons are sufficient to mediate ICSS reinforcement, potentially linking the MHb to the behavioral effects ascribed to tachykinins and their receptors.

In search of an optogenetic ICSS model, we examined reporter expression driven by a *Tac2^IRESCre^* transgenic line generated as part of the Allen Institute Transgenic Characterization Project (Harris et al., 2014). These mice were crossed with a Cre-dependent reporter line, *Ai32*, that conditionally expresses a ChR2-eYFP fusion protein (Madisen et al., 2012). Surprisingly, reporter expression more strongly resembled that of Tac1 mRNA than Tac2 mRNA, in that it was largely restricted to a subset of neurons in the dMHb (Fig. 5*C*). Expression of eYFP was rarely observed in the vMHb, as defined by the expression of the cholinergic marker choline acetyltransferase (Fig. 5*D*). PCR with allele-specific oligos confirmed the correct insertion of the targeted transgene at the *Tac2* locus. We note that Allen Institute database *in situ* hybridization data for *Tac2^IRESCre^* crossed with another reporter line, Ai14, shows the same pattern of reporter expression, restricted to the dMHb (Fig. 5*E*). Finally, we note that an independently generated *Tac2^Cre^* driver line, generated by replacement of the Tac2 gene rather than targeting of an IRES-Cre to the 3’-untranslated region of the transcript, shows a similar pattern of expression, primarily in the dMHb (Mar et al., 2012). Consistent with the pattern of marker expression driven by *Tac2^IRESCre^* in the dMHb, selective innervation by labeled fibers is found in the lateral nucleus of the IP (IPL; Fig. 5*F*–*H*), in which presynaptic dMHb fibers are marked by the expression of the *Tac1* gene product SP (Fig. 5*I*,*J*) and the *Tac2* gene product NKB (Fig. 5*K*). *Tac2^IRESCre^*-labeled fibers are relatively sparse in the rostral IP (IPR)/caudal IP (IPC) where Chat-immunoreactive fibers originating in the vMHb are located. It is not known why *Tac2^IRESCre^* shows a strong preference for the dMHb and its efferent target the IPL, but we conclude that *Tac2^IRESCre^* is a suitable model system for selective manipulation of tachykinin (Tac1/Tac2) expressing neurons in the dMHb.

**Figure 6.**
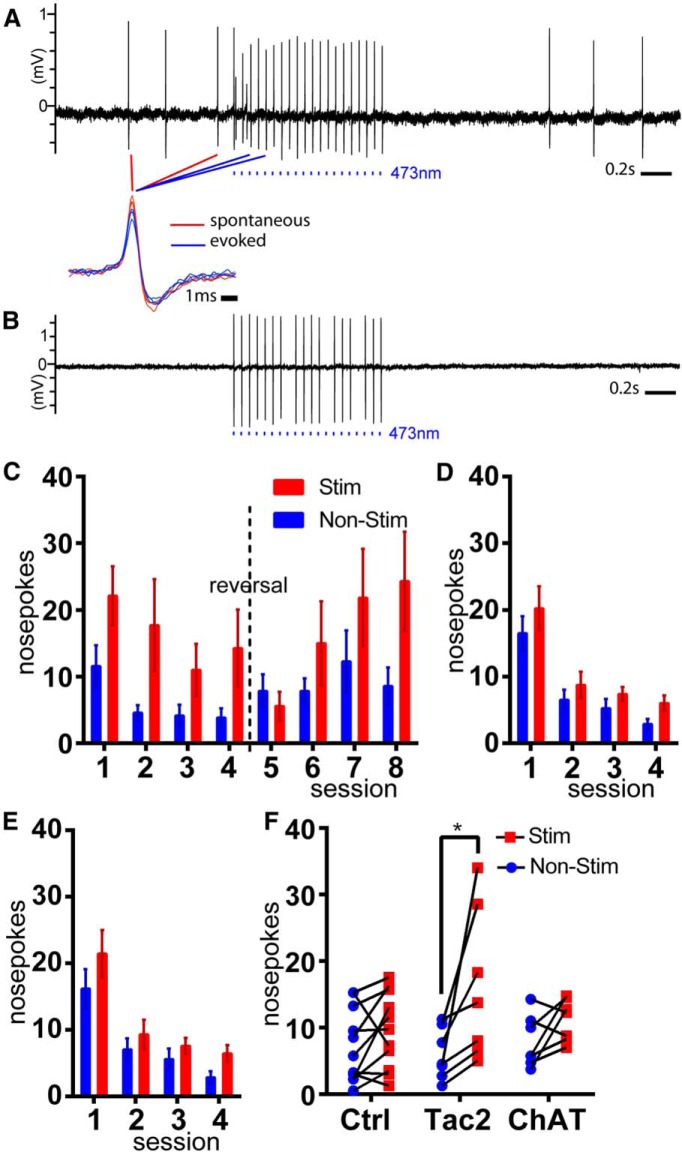
Intrinsic reinforcement mediated by neurokinin-expressing dMHb neurons. ***A***, ***B***, Light entrainment of dMHb neurons expressing Tac2^Cre^-driven ChR2. ***C***, Optogenetic ICSS in dMHb^ChR2^ mice. Two nose-poke receptacles in each behavioral compartment were randomized to active and inactive at the beginning of the trial. The initial assignment was maintained for days 1–4 of training, and then reversed for days 5–8 of training (reversal trials). Mice received a 2 s light stimulation of the dMHb for each nose-poke event in the active receptacle. ***D***, ICSS in control mice lacking a Cre-driver; no preference for the active receptacle was observed. ***E***, ICSS in vMHb^ChR2^ mice; no preference was observed. ***F***, Average values for nose-poke events in the inactive and active receptacles over 4 d of trials for dMHb^ChR2^ (*N*=7), vMHb^ChR2^ (*N*=6) and control (*N*=11) mice.

To test ChR2 function in *Tac2^IRESCre^*, *Ai32* (dMHb^ChR2^) mice, we first evaluated light-evoked neural activity in the dMHb using cell-attached recording in acute brain slices (Fig. 6*A*,*B*). Of the six light-responsive dMHb neurons sampled, one was spontaneously active at ∼4 Hz, one was spontaneously active at <1 Hz, and four were silent until exposed to light pulses. All six recorded neurons could be entrained with complete fidelity to a train of pulses delivered at 10 Hz; in some neurons entrainment with a 20 Hz pulse train resulted in omission of some spikes (Fig. 6*B*). The waveforms of spontaneous and evoked spikes were very similar (Fig. 6*A*, expanded view).

**Figure 7.**
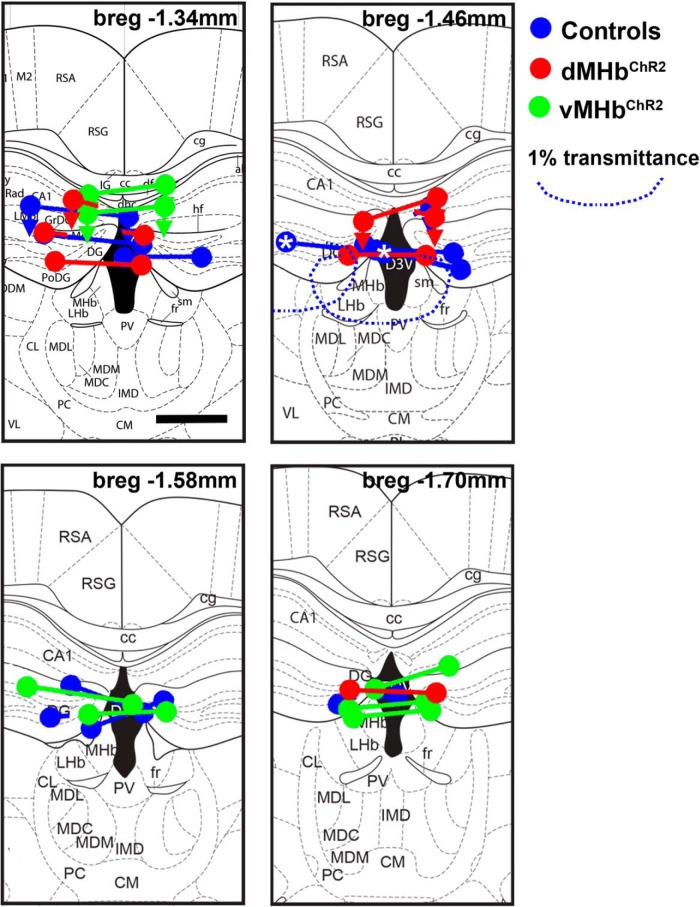
Placement of optical fibers in ICSS experimental mice. Mice with bilateral implanted fiber optic cannulas were perfused with a fixative at the conclusion of behavioral experiments and brains were examined for cannula placement. Fiber termini are shown on the level of a standard anatomical map (Paxinos and Franklin, 2001) closest to their rostrocaudal position at bregma −1.34, 1.46, 1.58, or 1.70 mm. Nearly all of the cannulas thus were positioned within ±0.2 mm of the intended coordinates at bregma −1.6 mm. Although some cannulas were displaced laterally, at least one of the two optical fibers terminated close to the habenula in every case. Fibers for dMHb^ChR2^ mice are shown in red, vMHb^ChR2^ mice are shown in green, and control mice are shown in blue. Connected dots indicate the probable ventral termini of the optical fibers from each case. In some cases, the right and left optical fibers mapped most accurately to different planes of section and are shown by disconnected dots. If the cannula track could not be followed to the terminus of the optical fiber, for instance due to termination in the ventricle, the most ventral position and the direction of the cannula track observed are indicated by an arrow. In all cases, the optical fibers were intact and transmitted light efficiently when examined postmortem after the experimental protocol. Scale bar, 0.5 mm. Light was delivered through the bilateral cannula for a total output of 8 mW, corresponding to 4 mW per 100 μm fiber or 509 mW/mm^2^ at the fiber teriminus. Because some of the cannulas were displaced laterally, we used a published empirically derived model for the diffusion of 473 nm light in the mouse brain tissue to estimate the light intensity at target structures (Yizhar et al., 2011). The example laterally displaced cannula pair is indicated by an asterisk (bregma 1.46 view). The boundary of light penetration at 1% of that delivered at the fiber terminus is indicated by a dashed line (∼5 mw/mm^2^). Although one cannula is displaced laterally, the medial cannula is predicted to illuminate the entire habenula. The predicted intensity of light, 5 mW/mm^2^, is sufficient to elicit reliable action potentials from dMHb neurons in brain-slice preparations.

To test whether dMHb neurokinin-expressing neurons mediate intrinsic reinforcement, we used an optogenetic ICSS protocol. dMHb^ChR2^ mice were implanted with a bilateral optical fiber cannula positioned just dorsal to the dMHb (Fig. 7). To determine whether vMHb neurons, identified by the expression of acetylcholine as a cotransmitter, might mediate ICSS reinforcement, we also implanted *Chat^Cre^*, *Ai32* (vMHb^ChR2^) mice. The neurons of vMHb^ChR2^ mice were previously shown to have light responses similar to those shown here for dMHb^ChR2^ neurons in acute brain slices (Hsu et al., 2013). Littermate mice lacking a Cre driver were implanted with optical fibers and used as controls for both optogenetic genotypes.

**Figure 8.**
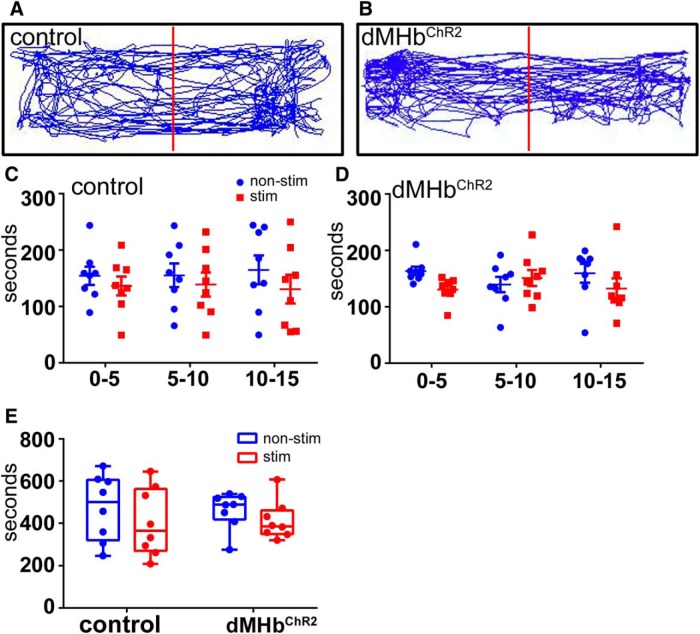
Real-time place preference. RTPP studies were conducted in a two-chamber place-preference box in which mice received light stimulation in one side, and could move freely between compartments. ***A***, ***B***, Example activity traces of control (***A***) and dMHb^ChR2^ (***B***) mice. Data are shown for an entire 15 min trial for a single animal of each genotype. ***C***, ***D***, Side preference of control (***C***; *n*=8) and dMHb^ChR2^ mice (***D***; *n*=8) displayed in 5 min bins over the course of a 15 min trial. The large variability in the side occupancy in the later intervals is likely to represent decreasing exploration during the course of the trial, with individual mice settling on one side of the chamber or the other, apparently without preference. ***E***, Summary of side occupancy for 15 min trial. No significant effect of side or genotype was observed.

ICSS was evaluated in four training sessions in which mice were presented with a two nose-poke receptacles, one of which delivered a 2 s train of light pulses upon entry, and one of which was inactive. dMHb^ChR2^ mice developed a preference for the active receptacle on the first training day, which persisted through 4 d of training (Fig. 6*C*,*F*). The cohort of dMHb^ChR2^ mice then underwent four sessions in which the active receptacle was reversed. One day (day 5) was required for extinction of the response to the previously active receptacle, and by day 6 of training, a preference for the newly active receptacle was established. In contrast, vMHb^ChR2^ mice (Fig. 6*D*,*F*) and control mice (Fig. 6*E*,*F*) did not establish a significant preference over 4 d of training. ICSS using a nose-poke response is not an effective measure of aversion, because both a neutral stimulus and an aversive one may elicit no response. For this reason, we tested possible aversion to light stimulation in dMHb^ChR2^ mice in a real-time place preference paradigm (Hsu et al., 2014). No aversive or reinforcing response to dMHb stimulation was detected in this paradigm (Fig. 8). We conclude that the reinforcing effect of MHb stimulation is specifically conferred by activation of neurokinin-expressing dMHb neurons, and that ICSS is more effective at detecting this effect than place preference.

## Discussion

Fear conditioning, the induction and learning of threat responses in rodents, is a widely used model of human stress-related disorders. Studies of the neural pathways underlying contextual and cued (Pavlovian) fear conditioning have focused on core circuitry involving the amygdala (Dejean et al., 2015; Keifer et al., 2015), with the integration of hippocampal and frontal circuits in contextual conditioning (Maren et al., 2013; Rozeske et al., 2015), and the periaqueductal gray in mediating the freezing response. Although the dMHb is not part of this amygdala-centered circuitry known to be involved in conditioned fear, a recent report suggests involvement of the septohabenular pathway in fear behavior (Yamaguchi et al., 2013). In this study, immunotoxin-mediated cell targeting was used to ablate neurons in the bed nucleus of the anterior commissure (BAC), part of the septal complex, that project specifically to the dMHb. BAC-ablated mice showed increased freezing response during fear training, but the effect on conditioned fear was not reported. Here we have used a contextual conditioning protocol, without a specific cue, to reproduce the original study to the extent possible. Our results demonstrate that the dMHb is not essential for the acquisition of acute fear or for the contextual conditioned fear response. Further understanding of the septohabenular circuitry may shed light on why the fear response was not affected in our study. It is possible that ablation of the BAC may have effects on fear behavior that are not mediated by the dMHb.

Consistent with our results, lesions of the entire habenula (MHb + LHb) in mice show no net effect on conditioned fear (Heldt and Ressler, 2006). However, a recent study has shown that cue-conditioned fear is modulated by cannabinoid receptors in the MHb (Soria-Gómez et al., 2015). This effect is attributed to vMHb neurons that use acetylcholine as a cotransmitter, which are intact in the mice used in the present study (Fig. 1) rather than the dMHb. Thus, to date there is no evidence for a direct role for the dMHb in fear conditioning.

Here and in a prior study (Hsu et al., 2014), we have examined the effect of dMHb ablation in several widely-used models of depression, including the Porsolt FST, the TST, the sucrose preference test, and learned helplessness. Ablation of the dMHb had no discernable effect on the FST (Hsu et al., 2014), yet in the present study, dMHb^CKO^ mice show reduced immobility in the conceptually-related TST, a result that could be interpreted as an antidepressant response. These tests are conceptually associated based on the common concept of “behavioral despair”, originally applied to the FST, in which rodents initially attempt to escape an adverse situation, and then become passive and relatively immobile. The widely used animal models of depression have been linked to mood states by their face validity and their value in predicting antidepressant responses (Cryan and Mombereau, 2004; Duman, 2010). However, antidepressants work on neurotransmitter systems with broad CNS effects. Because the FST and TST involve different stimuli and different locomotor responses, there is no reason to assume that these tests will always have concordant results in transgenic models that affect specific neural pathways. Supporting this concept, quantitative trait locus analysis of mouse strains that show differential behavior in the TST and FST have mapped both distinct and common gene loci linked to behavior in these tests (Yoshikawa et al., 2002; Tomida et al., 2009).

One strain of genetically altered mice shown to have decreased immobility time (antidepressant effect) in the TST and FST are those with a specific deletion of the *Tac1* gene, encoding the neuropeptides SP and neurokinin A (Bilkei-Gorzo et al., 2002). Tac1 mRNA, expression of which distinguishes the dMHb from the adjacent vMHb and LHb, is nearly absent in dMHb^CKO^ mice (Hsu et al., 2014). SP is also strongly expressed in dMHb fibers terminating in the lateral part of the interpeduncular nucleus in the ventral midbrain (Hsu et al., 2013). Thus, it is possible that the decreased TST immobility time observed in dMHb^CKO^ mice results from a loss of *Tac1* peptide signaling in the habenulopeduncular system; the effect of *Tac1* gene deletion on the FST may reside in another pathway. NKB, the *Tac2* gene product coexpressed with SP in the dMHb and its terminal fibers in the IP, may also play a role in these effects, but little is known about the specific function of this peptide in the CNS. dMHb^CKO^ mice show marked sensitization to the induction of learned helplessness. In contrast, ablation of the entire habenula attenuates the learned helplessness response (Amat et al., 2001), an effect which may be attributable to the LHb, because the induction of learned helplessness increases synaptic inputs to LHb neurons (B. Li et al., 2011). We expected to find that the sensitization to learned helplessness in dMHb^CKO^ mice resulted from the marked loss of dMHb neurons in these animals. However, Pou4f1^+/-^ mice, in which the dMHb is intact, also show sensitization to the induction of learned helplessness, demonstrating that *Pou4f1* haploinsufficiency is sufficient to produce this effect. None of the other behavioral paradigms tested were affected by *Pou4f1* haploinsufficiency.

The effect of *Pou4f1* haploinsufficiency on learned helplessness was not anticipated in the light of prior work on the role of this transcription factor in neural development and gene regulation. Independent null alleles of *Pou4f1* have been generated in at least three laboratories. Homozygous *Pou4f1* null mutants die shortly following birth, probably from defects in brainstem motor systems (McEvilly et al., 1996), but *Pou4f1* hemizygous mice are viable, fertile, and have no known developmental defects (McEvilly et al., 1996; Xiang et al., 1996; Quina et al., 2005). Furthermore, in the peripheral sensory nervous system, where Pou4f1 is a key developmental regulator, an autoregulatory mechanism has been identified which compensates for *Pou4f1* gene dosage in heterozygous null embryos, nearly normalizing the expression of downstream regulatory targets (Trieu et al., 2003; Eng et al., 2004). Pou4f1 is expressed in multiple CNS regions that are potential candidates for mediating the effect of haploinsufficiency on learned helplessness. These include the lateral habenula, superior colliculus (tectum), interpeduncular nucleus, red nucleus, and inferior olive (Fedtsova and Turner, 1995). Aside from the lateral habenula, however, the effect of these brain regions on mood regulation and stress response is not well characterized.

In a prior study, we have shown that dMHb^CKO^ mice have markedly reduced voluntary wheel running activity (Hsu et al., 2014). This appears to be a specific deficit in exercise reinforcement, since basal locomotion is not affected. Although not generally used as a model of depression, WRA may interact with affective state and has been shown to produce an antidepressant-like effect in rats and mice (Greenwood et al., 2003; Duman et al., 2008). Thus, we conclude that the dMHb circuit clearly intersects pathways for depression-related behaviors across multiple models. These results are broadly consistent with the role of the dMHb in exercise reinforcement and intracranial self-stimulation reinforcement. However, ablation of the dMHb does not simply cause depression-related behaviors, nor does it prevent them in a way that encompasses all of the behavioral constructs used to model mood disorders and fear. As specific tools are used to dissect the underlying brain circuits for each of these mood-related behaviors, these pathways may be shown to impact depression-related behaviors by distinct mechanisms.

## Synthesis

The decision was a result of the Reviewing Editor Daniela Schiller and the peer reviewers coming together and discussing their recommendations until a consensus was reached. A fact-based synthesis statement explaining their decision and outlining what is needed to prepare a revision is listed below. The following reviewer(s) agreed to reveal their identity: Vijay Mohan Namboodiri

The study presents a series of studies assessing the role the dorsal medial habenula (dMHb) plays in in depression-like behavior, reinforcement and fear. The authors target the dMHb by using a mousseline in which the gene for the dMHb/LHb specific transcription factor Pou4f1 has been 'floxxed'. When cre recombinase is put under the control of the dMHb specific promoter Syt6, a dMHb specific lesion is achieved. The animals are then put through a series of behavioral tests. Finally, optogenetic techniques are used to isolate some of the dMHb ascribed behaviors to a specific cell-type, namely neuokinin expressing neurons. The strength of the paper is in using some innovative transgenic and optogenetic strategies to ascribe behavioral output to a small population cells within the habenular structure. However, in its current form the findings from the manuscript are somewhat disjointed. Furthermore, some of the most interesting findings are not further investigated.

Major Points

1) One of the main issues with the manuscript is that the results from many of the tests are somewhat contradictory. For example, in the present manuscript and in a previous report by the same authors (Hsu et al., 2014) the authors repeatedly state the CKO mice have deficits in locomotor tasks when a motivational component is present. This would seem to line up with the learned helplessness data presented but is at odds with the tail suspension test data - reduced immobility in the TST is indicative of increased escape-focused orientated behavior which is the opposite to the increased learned helplessness seen in the CKO animals. The authors do show that the Pou4f1 haploinsuffiency may be at the root of the increase to learned helplessness. Is it likely that the previous data, which forms the basis for much of the assumptions made in this report, e.g. the observed anhedonia may in fact be due to the Pou4f1 haploinsuffiency? Indeed, the authors state they find that the wheel running phenotype is not due to Pou4f1 haploinsuffiency but that is being prepared for another submission. However, currently this data is not published and is not in the current manuscript and as such cannot be judged by a reviewer and inform the basis for the current conclusions.

2) Manipulation of dMHb need not necessarily produce consistent results between different assays of depression. However, it needs to be discussed how the lesion of neurons promoting a rewarding behavior produces antidepressant effects. One would imagine that lesion of neurons mediating reward would result in depressant effects. The story would be much easier to interpret if a cell-type specific inhibition targeting the Tac2 expressing cells from the optogenetic experiment is performed. To this end, the authors could combine a purely genetic strategy (Tac2-Cre line crossed with a Cre-inducible hM4Di receptor line) with local infusion of CNO to limit the spread of inactivation (the Allen ISH atlas shows that there aren't any nearby regions that express those genes and of course, a systemic injection of CNO would not work). It is not necessary to perform this experiment but recommended as it might make the data much easier to interpret.

3) Have the authors assessed if sensitivity to noxious stimuli are altered in these CKO animals? this is a caveat which needs to be considered when interpreting behavior induced via a foot shock.

4) The authors describe that the CKO mice have reduced immobility in the TST and this is at odds with the data previously obtained in the FST. As the authors point out is indeed true that these tests do not always produce the same result, however there may not be a discrepancy in this case. The published FST data looks to have been analyzed in an unorthodox fashion. Usually the cumulative immobility time of the final 4 minutes of a 6 minutes are scored (Cryan and Holmes 2005). However, the authors seemed to have looked at the data over 14 minutes of testing. Looking at the graph it looks like there may be a reduction in immobility over this 4-minute period in the CKO animals. This would further complicate the findings given the anhedonia like phenotype described previously. Again, is it possible some of the behavioral disparity may be due to Pouf4f1 haplo insuffincey and not the DMHb CKO per se?

5) A major gap in the paper is that the authors have isolated the reinforcing effects of DMHb stimulation to a specific cell-type i.e. neurokinin expressing neurons and have not then assessed if the other behaviors affected by CKO e.g. the TST can be isolated to these neurons. Isolating the behaviors described here and in their previous publication to either neurokinin expressing neurons or Pou4f1 haploinsufficiecy may strengthen the impact of the manuscript.

6) When the aim is to assess whether the effect of dMHb lesion can be explained by Pou4f1 haploinsufficiency, the appropriate test is to compare the effect of lesion to that of haploinsufficiency. So the test would be to compare the difference between experimental and control groups for the lesion and haploinsufficiency experiments to each other. It is likely that the current conclusions will stand after performing the appropriate comparisons but it is still important to do it correctly.

7) Multiple comparisons correction is not necessary in studies running a battery of tests just due to selective presentation of results. In the absence of specific null hypotheses for each behavioral test, the likelihood of observing a significant effect for any one of them increases. Of course, if the authors started out with specific hypotheses like "dMHb lesion should have a specific effect on TST but not FST", no such correction is required. In the absence of such a specific hypothesis, an effect in any one test would be deemed as a "significant" effect, increasing the probability of Type I errors. So in order to perform correct inference, such multiple comparisons correction is indeed necessary (it is very likely that the results will hold up after multiple comparisons).

8) It is plausible that dMHb need not necessarily produce consistent results between different assays of depression. However, it needs to be discussed how the lesion of neurons promoting a rewarding behavior produces antidepressant effects. One would imagine that lesion of neurons mediating reward would result in depressant effects. The other "inconsistencies" here will be easier to interpret if this was clarified.

9) When statements regarding the function of LHb are made, it is important to cite the relevant literature. This issue is raised primarily because of the summary statement of LHb function as being involved in the expression of learned helplessness. This is an oversimplification that largely misses the much more well established role of LHb in reward and aversive functioning.

10) In figure 8 it might be more clear if the authors kept the same nomenclature as in figure 6 and refer to the chambers as 'stim' and 'non-stim' instead of active and inactive.
